# Supportive care in patients with advanced non-small-cell lung cancer

**DOI:** 10.1038/sj.bjc.6601236

**Published:** 2003-09-09

**Authors:** M Di Maio, F Perrone, C Gallo, R V Iaffaioli, L Manzione, F V Piantedosi, S Cigolari, A Illiano, S Barbera, S F Robbiati, E Piazza, G P Ianniello, L Frontini, E Veltri, F Castiglione, F Rosetti, E De Maio, P Maione, C Gridelli

**Keywords:** supportive care, lung cancer, polypharmacotherapy, concomitant drugs

## Abstract

The present study describes supportive care (SC) in patients with advanced non-small-cell lung cancer (NSCLC), evaluating whether it is affected by concomitant chemotherapy, patient's performance status (PS) and age. Data of patients enrolled in three randomised trials of first-line chemotherapy, conducted between 1996 and 2001, were pooled. The analysis was limited to the first three cycles of treatment. Supportive care data were available for 1185 out of 1312 (90%) enrolled patients. Gastrointestinal drugs (45.7%), corticosteroids (33.4%) and analgesics (23.8%) were the most frequently observed categories. The mean number of drugs per patient was 2.43; 538 patients (45.4%) assumed three or more supportive drugs. Vinorelbine does not produce substantial variations in the SC pattern, while cisplatin-based treatment requires an overall higher number of supportive drugs, with higher use of antiemetics (41 *vs* 27%) and antianaemics (10 *vs* 4%). Patients with worse PS are more exposed to corticosteroids (42 *vs* 30%). Elderly patients require drugs against concomitant diseases significantly more than adults (20 *vs* 7%) and are less frequently exposed to antiemetics (12 *vs* 27%). In conclusion, polypharmacotherapy is a relevant issue in patients with advanced NSCLC. Chemotherapy does not remarkably affect the pattern of SC, except for some drugs against side effects. Elderly patients assume more drugs for concomitant diseases and receive less antiemetics than adults.

Data from meta-analysis (Non-small Cell Lung Cancer Collaborative Group, 1995) show a slight but significant median survival advantage (6 weeks) for platinum-containing chemotherapy in the treatment of patients with advanced non-small-cell lung cancer (NSCLC). Some benefit was also shown for chemotherapy in elderly patients, in terms of both survival and quality of life (The ELVIS group, 1999).

Based on these results, although the efficacy of currently available chemotherapy is far from being satisfactory, the majority of patients diagnosed with advanced NSCLC are offered chemotherapy. In addition to antineoplastic drugs, a variable number of different drugs are given as supportive care (SC), that is, ‘*every treatment given to prevent, control or relieve complications and side effects and to improve the patient's comfort and quality of life of people who have cancer*’ (National Cancer Institute Dictionary, 2002).

The concomitant assumption of several drugs leads to obvious pharmacoeconomic and practical problems of compliance for patients, and raises important safety issues. Pharmacological interactions are identified as one of the eight ‘drug-related problems’ (Strand *et al*, 1990) and are a common and serious consequence of polypharmacotherapy (Cadieux, 1989). At a pharmacokinetic level, one drug can interfere with absorption, distribution, metabolism and excretion of another drug. Moreover, the assumption of a high number of drugs can reduce patients' compliance and, more dangerously, induce errors of dose and timing of assumption (Hulka *et al*, 1975). All of these problems can give rise to toxic effects, especially when drugs with a low therapeutic index, like cytotoxic drugs, are used.

The present study has two main aims: first, to give a picture of the number and variety of supportive drugs assumed by patients with advanced NSCLC during chemotherapy, and, second, to assess the impact of chemotherapy, patient's performance status (PS) and patient's age on the assumption of SC.

## MATERIALS AND METHODS

### Patients

Patients with advanced NSCLC who participated in three randomised clinical trials (The ELVIS group, 1999; Gridelli *et al*, 2003a, 2003b) performed by our cooperative group between 1996 and 2001 were selected for this study. All three studies were approved by Ethical Committees, and all patients gave written informed consent. They had stage IV or IIIB (with supraclavicular metastatic nodes or malignant pleural effusion) disease and a baseline PS not worse than 2, according to the ECOG scale (Oken *et al*, 1982).

In the ELVIS study (Elderly) Lung cancer Vinorelbine Italian Study (The ELVIS group, 1999), vinorelbine was compared with SC alone in patients ⩾70 years. Vinorelbine was given 30 mg m^−2^ on days 1 and 8 every 3 weeks, for six cycles. The primary end point was quality of life. Recruitment started in April 1996, and overall 191 patients were randomised.

The MILES study (Multicentre Italian Lung cancer in the Elderly Study (Gridelli *et al*, 2003b)) was conducted in the same subset of patients of the ELVIS trial, and compared the combination of vinorelbine and gemcitabine *vs* the two single drugs. Patients were randomly assigned vinorelbine (30 mg m^−2^), gemcitabine (1200 mg m^−2^) or vinorelbine (25 mg m^−2^) plus gemcitabine (1000 mg m^−2^). All treatments were delivered on days 1 and 8 every 3 weeks for six cycles. The primary end point was overall survival. In all, 707 patients were randomised between December 1997 and November 2000.

The GEMVIN3 study (Gridelli *et al*, 2003a) was conducted with adult (<70 years) patients, randomly assigned vinorelbine (25 mg m^−2^, days 1 and 8) plus gemcitabine (1000 mg m^−2^, days 1 and 8) or cisplatin-based chemotherapy: cisplatin (80 mg m^−2^, day 1) either plus gemcitabine (1200 mg m^−2^, days 1 and 8) or plus vinorelbine (30 mg m^−2^, days 1 and 8) for six cycles of 21 days. The study aimed to assess whether the combination of gemcitabine and vinorelbine improved quality of life, without shortening survival, compared to standard platinum-containing regimens. Accrual started in Italy in October 1998, and in Canada in May 1999. Overall, 503 patients (414 in Italy) were randomised, between October 1998 and March 2001. In this analysis, only Italian patients are considered.

### Protocol requirements and data collection on SC

In all the three trials, investigators were free to choose SC. Each protocol reported only general guidelines about the modalities of administration of the main categories of supportive drugs.

The three-step WHO ladder (World Health Organization, 1996) was recommended for treatment of pain. In case of febrile infections, the association of a third-generation cephalosporin and an aminoglycoside was recommended, with modifications determined by the results of haemoculture. In case of grade 4 neutropenia, prophylactic administration of a quinolone was recommended and use of colony-stimulating factors (CSF) was allowed until resolution of toxicity. No prophylactic administration of CSF was mandated by protocol. Corticosteroids were recommended for hypercalcaemia, respiratory failure and intracranial hypertension.

The three studies had the same Case Report Form for SC. Data were collected from the starting date of chemotherapy until interruption, for up to seven drugs in each cycle of chemotherapy (corresponding to a theoretic 21-day period), with the daily dose and the number of days of assumption recorded. Importantly, drugs administered as premedication before chemotherapy (e.g. antiemetics, diuretics) did not have to be reported in the SC CRF.

All drugs assumed by patients have been coded according to the anatomical therapeutic chemical (ATC) classification system (World Health Organization Collaborating Centre for Drug Statistics Methodology, 2002). This system has been recommended for international studies on drug consumption by the WHO Collaborating Centre for Drug Statistics Methodology. In the ATC system, the drugs are divided into different groups according to the system on which they act and their chemical, pharmacological and therapeutic properties. Drugs are classified at five different levels: 14 main groups (first level), two therapeutic/pharmacological subgroups (second and third levels), a therapeutic/pharmacological/chemical subgroup (fourth level) and the chemical substance (fifth level).

### Analysis

In all the three studies analysed, instrumental restaging of the patients was planned after three cycles of chemotherapy, and patients with progressive disease did not receive any more protocol treatment. So, in order to reduce the amount of missing data and possible selection biases, analyses have been limited to the period of time corresponding to the first three cycles (theoretically 63 days). The number of drugs assumed by each patient and frequencies within ATC categories were used for analyses. The whole sample was taken into account for description of the SC pattern. No analysis was carried out on dose and frequency of drug assumption.

For comparative purposes, supportive drugs were grouped into three categories, to reduce the number of statistical comparisons:

1. Drugs used against side effects of treatment: stomatologicals, antiacids, antispasmodics, antiemetics, laxatives, antidiarrhoeals, antihaemorragics, antianaemics, anti-infectives, CSF.
2. Drugs used against tumour-related symptoms: anabolic agents, appetite stimulants, antithrombotics, ACTH and corticosteroids, progestogens, anti-inflammatory drugs and analgesics, drugs affecting demineralisation, antiepileptics, antiasthmatics, expettorants and cough suppressants.
3. Drugs assumed against concomitant diseases: antidiabetics, drugs for cardiovascular system, drugs for thyroid, drugs for benign prostatic hypertrophy, psycholeptics and psychoanaleptics.

This grouping procedure was somewhat arbitrary for some categories (e.g. respiratory drugs could have been prescribed because of concomitant chronic obstructive pulmonary disease rather than tumour symptoms). Vitamins, integrators and mineral supplements, which are not reasonably attributable to any category, were described but not considered in statistical comparisons.

Four questions were addressed in four different subgroups of patients:

1. Does chemotherapy affect SC? – addressed in patients randomised in the ELVIS trial (vinorelbine *vs* SC) (The ELVIS group, 1999).
2. Does cisplatin-based chemotherapy affect SC? – addressed in patients randomised in the GEMVIN3 study (cisplatin-based *vs* noncisplatin-based chemotherapy) (Gridelli *et al*, 2003a).
3. Does performance status affect SC? – addressed comparing patients with PS 0/1 *vs* patients with PS 2 from all the three studies (The ELVIS group, 1999; Gridelli *et al*, 2003a, 2003b). Owing to the worse prognosis of PS2 patients and the corresponding reduced number of cycles of chemotherapy, we limited this analysis only to patients who had fulfilled all the three first cycles, in the attempt of not underestimating the assumption of supportive drugs among PS 2 subjects.
4. Does age affect SC? – addressed comparing patients receiving gemcitabine plus vinorelbine in the GEMVIN3 study (Gridelli *et al*, 2003a) (adults) *vs* those receiving the same chemotherapy in the MILES study (Gridelli *et al*, 2003b) (elderly).

Within randomised comparisons (questions 1 and 2), differences in the number of assumed drugs were tested by the Wilcoxon rank-sum test and differences in the use of different groups of drugs were tested by *χ*^2^ test. Two-sided *P*-values less than 0.05 were considered statistically significant. Questions 3 and 4 used patients from different studies, thus some adjustment was needed. A possible confounding effect, indeed, could arise from enrollment bias among studies when assessing the role of PS and from a different distribution of PS between adult and elderly subjects when assessing the role of age. Thus, stratified Wilcoxon rank-sum test and Mantel–Haenszel test (Mantel and Haenszel, 1959) were carried out after stratification by treatment arm and PS category, respectively (StatXact^©^ turbo, CYTEL software Corp. Cambridge, MA, USA, 1992). Homogeneity assumption among strata was previously tested by the Breslow–Day test (Breslow and Day, 1980).

## RESULTS

Data on SC were available in 165 out of 191 patients (86%) in the ELVIS study, in 655 out of 707 (93%) in the MILES study and in 365 out of 414 (88%) in the GEMVIN3 study. Overall, data were available for 1185 patients out of the 1312 (90%) enrolled. Principal characteristics of the 1185 analysed patients, according to treatment arm, are described in Table 1

**Table 1:** Characteristics of the patients for whom information on supportive care (SC) was available, divided by trial and chemotherapy arm

**Study**	**ELVIS trial**		**MILES trial**			**GEMVIN3 trial**		
**Arm**	**SC alone**	**Vinorelbine**	**Vinorelbine**	**Gemcitabine**	**Vinorelbine+gemcitabine**	**Vinorelbine+gemcitabine**	**CDDP-based chemotherapy**	**All patients**
Median age (range)	74 (69–85)	74 (69–85)	73 (64–83)	73 (69–86)	73 (69–84)	63 (37–70)	64 (36–71)	71 (36–86)
Gender								
Male	80 (89%)	65 (87%)	192 (87%)	179 (83%)	173 (79%)	150 (82%)	153 (85%)	992 (84%)
Female	10 (11%)	10 (13%)	28 (13%)	37 (17%)	46 (21%)	34 (18%)	28 (15%)	193 (16%)
PS								
0	17 (19%)	15 (20%)	66 (30%)	64 (30%)	60 (27%)	56 (30%)	55 (30%)	333 (28%)
1	52 (58%)	41 (55%)	113 (51%)	113 (52%)	120 (55%)	103 (56%)	102 (56%)	644 (54%)
2	21 (23%)	19 (25%)	41 (19%)	39 (18%)	39 (18%)	25 (14%)	24 (13%)	208 (18%)
								
No of patients	90	75	220	216	219	184	181	1185

. The median age was 74 years (range 69–85) in the ELVIS study, 73 years (64–86) in the MILES study and 63 years (36–71) in the GEMVIN3 study. Most of the patients were males (88, 83 and 83%, in the ELVIS, MILES and GEMVIN3 trial, respectively). Performance status was equal to 0 in 19, 29 and 30%, equal to 1 in 56, 53 and 56%, and equal to 2 in 24, 18 and 13%, in ELVIS, MILES and GEMVIN3, respectively.

### Description of SC

A total of 382 different products (corresponding to 265 different active principles), representing 13 of the 14 ATC main groups, were assumed: 136 mainly directed against treatment side effects (95 active principles), 137 against tumour symptoms (88 active principles) and 109 against concomitant diseases (82 active principles). In addition, 21 products were vitamins, integrators and mineral supplements.

**Figure 1:**
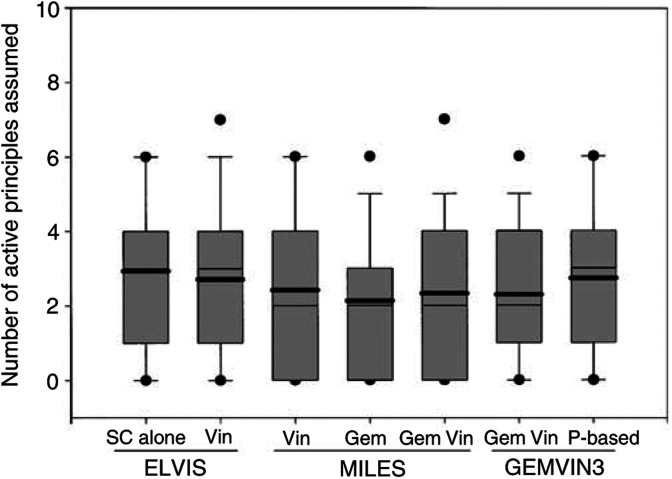
Number of active principles assumed in different treatment arms. Thiner and thicker line in the box: median and mean. Box hinges: 25–75th percentiles; ends of the segments: 10–90th percentiles; dots: 5–95th percentiles. SC=supportive care; Vin=vinorelbine; Gem=gemcitabine; P=cisplatin.

Polypharmacotherapy was frequent: patients assumed 2.43 drugs on average; 898 (75.8%) assumed at least one supportive drug, 709 (59.8%) two or more, 538 (45.4%) three or more. The number of drugs assumed was similar across different treatment arms (Figure 1

).

The number of patients for the main ATC categories and some subcategories are reported in Table 2

**Table 2:** Drugs assumed by the 1185 patients, classified according to the WHO ATC system

**ATC code**	**Description**	**Category**	**No. of pts.**	**(%)**
A	*Alimentary tract and metabolism*		*542*	*(45.7)*
A01	Stomatologicals	SE	9	(0.8)
A02	Antacids and drugs for treatment of peptic ulcer	SE	245	(20.7)
A03	Antispasmodic, anticholinergics and propulsives	SE	148	(12.5)
A04	Antiemetics and antinauseants	SE	193	(16.3)
A06	Laxatives	SE	116	(9.8)
A07	Antidiarrhoeals and intestinal anti-infective agents	SE	31	(2.6)
A10	Drugs used in diabetes	CD	18	(1.5)
A11	Vitamins	–	71	(6)
A12	Mineral supplements	–	4	(0.3)
A14	Anabolic agents for systemic use	TS	12	(1)
A15	Appetite stimulants	TS	1	(0.1)

B	*Blood and blood-forming organs*		*150*	*(12.7)*
B01	Antithrombotic agents	TS	35	(3)
B02	Antihaemorrhagics	SE	43	(3.6)
B03	Antianaemic preparations	SE	54	(4.6)

C	*Cardiovascular system*	*CD*	*141*	*(11.9)*

D	*Dermatologicals*	CD	3	(0.3)

G	*Genitourinary system and sex hormones*		4	(0.3)
G04	Drugs for benign prostatic hypertrophy	CD	4	(0.3)

H	*Systemic hormonal preparations*		*420*	*(35.4)*
H01AA	ACTH	TS	39	(3.3)
H02	Corticosteroids for systemic use	TS	396	(33.4)
H03	Thyroid therapy	CD	3	(0.2)

J	*General anti-infectives for systemic use*		*126*	*(10.6)*
J01	Antibacterials for systemic use	SE	114	(9.6)
J02	Antimycotics for systemic use	SE	20	(1.7)

L	*Antineoplastic and immunomodulating agents*		*113*	*(9.5)*
L02AB	Progestogens	TS	18	(1.5)
L03AA	Colony-stimulating factors	SE	97	(8.2)

M	*Muscoloskeletal agents*		*222*	*(18.7)*
M01A	Nonsteroidal anti-inflammatory drugs	TS	200	(16.9)
M05B	Drugs affecting mineralisation	TS	34	(2.9)

N	*Nervous system (inch. Analgesics)*		*282*	*(23.8)*
N02A	Opioids	TS	117	(9.9)
N02B	Other analgesics and antipyretics	TS	143	(12.1)
N03	Antiepileptics	TS	6	(0.5)
N05	Psycoleptics	CD	36	(3)
N06	Psycoanaleptics	CD	15	(1.3)
N07C	Antivertigo preparations	CD	2	(0.2)

P	*Antiparasitic products*	*CD*	*0*	–

R	*Respiratory system*		*195*	*(16.5)*
R03	Antiasthmatics	TS	95	(8)
R05C	Expettorants	TS	47	(4)
R05D	Cough suppressants	TS	90	(7.6)

S	*Sensory organs*	*CD*	*3*	*(0.3)*

V	*Various*	–	*23*	*(1.9)*

SE=against side effects of treatment; TS=against tumour symptoms; CD=against concomitant diseases.

. Gastrointestinal drugs (A, 45.7%), corticosteroids (H02, 33.4%), analgesics (N, 23.8%), anti-inflammatory drugs (M, 18.7%) and drugs for respiratory system (R, 16.5%) were the most frequently observed ATC codes.

Overall, 680 (57.4%), 633 (53.4%) and 199 (16.8%) patients assumed at least one drug against tumour symptoms, treatment side effects and concomitant diseases, respectively.

### Does chemotherapy affect SC?

In the ELVIS study, comparing vinorelbine *vs* SC alone (Table 3

**Table 3:** Does chemotherapy affect supportive care (SC)?

	**SC alone (*n*=90)**	**Vinorelbine (*n*=75)**	***P*^a^**
*Drugs against treatment side effects*	*41 (46%)*	*35 (47%)*	*0.89*
A01 Stomatologicals	0	0	
A02 Antacids	29 (32%)	14 (19%)	
A03 Antispasmodics	4 (4%)	5 (7%)	
A04 Antiemetics	0	2 (3%)	
A06 Laxatives	4 (4%)	6 (8%)	
A07 Antidiarrhoeals	1 (1%)	1 (1%)	
B02 Antihaemorrhagics	4 (4%)	7 (9%)	
B03 Antianaemics	1 (1%)	3 (4%)	
J01 Antibacterials for systemic use	10 (11%)	6 (8%)	
J02 Antimycotics for systemic use	0	1 (1%)	
L03AA Colony-stimulating factors	0	5 (7%)	

*Drugs against tumour symptoms*	*66 (73%)*	*53 (71%)*	*0.70*
A14 Anabolic agents for systemic use	3 (3%)	3 (4%)	
A15 Appetite stimulants	1 (1%)	0	
B01 Antithrombotic agents	0	1 (1%)	
H01AA ACTH	7 (8%)	0	
H02 Corticosteroids for systemic use	45 (50%)	32 (43%)	
L02AB Progestogens	2 (2%)	3 (4%)	
M01A Nonsteroidal anti-inflammatory drugs	26 (29%)	14 (19%)	
M05B Drugs affecting mineralisation	5 (6%)	1 (1%)	
N02A Opioids	16 (18%)	9 (12%)	
N02B Other analgesics and antipyretics	12 (13%)	8 (11%)	
N03 Antiepileptics	0	0	
R03 Antiasthmatics	8 (9%)	5 (7%)	
R05C Expettorants	10 (11%)	9 (12%)	
R05D Cough suppressants	14 (16%)	11 (15%)	

*Drugs against concomitant diseases*	*27 (30%)*	*17 (23%)*	*0.29*
A10 Antidiabetics	1 (1%)	0	
C Cardiovascular system	17 (19%)	12 (16%)	
G04 Drugs for prostatic hypertrophy	1 (1%)	0	
H03 Drugs for thyroid	0	0	
N05 Psycholeptics	8 (9%)	5 (7%)	
N06 Psychoanaleptics	2 (2%)	0	
N07C Antivertigo preparations	1 (1%)	0	

Elderly patients randomised in the ELVIS trial. The table shows the number (percentage) of patients assuming at least one drug of each category during the first 63 days of treatment.

a *χ*^2^ test.

), 131 out of 165 patients (79%) assumed at least one supportive drug. The mean number of supportive drugs assumed in the vinorelbine arm was 2.5 as compared with 2.8 in the SC alone arm (*P*=0.22). Drugs against side effects of treatment were assumed by 46% of patients in SC arm and 47% of those in the vinorelbine arm (*P*=0.89); this result is largely driven by antiacids presumably prescribed to counteract gastric toxicity of corticosteroids and other anti-inflammatory drugs. Drugs against symptoms were assumed by 73% in the SC arm and 71% in the vinorelbine arm (*P*=0.70); drugs against concomitant diseases were assumed by 30% in the SC arm and 23% in the vinorelbine arm (*P*=0.29). As expected, haemopoietic growth factors were assumed only in the chemotherapy arm, by 7% of patients.

### Does cisplatin-based chemotherapy affect SC?

In the GEMVIN3 study, comparing cisplatin-based chemotherapy with gemcitabine plus vinorelbine, 288 out of 365 patients (79%) assumed at least one supportive drug. The mean number of supportive drugs was higher in the cisplatin arm (2.6 *vs* 2.2, *P*=0.055). Drugs against treatment side effects were assumed by 66 and 61% of the patients (*P*=0.33), drugs against symptoms by 57 and 53% (*P*=0.42) and drugs against concomitant disease by 11 and 7% (*P*=0.13), in the cisplatin and in the non-cisplatin arm, respectively. As expected, relevant differences were observed for antiemetics (41 *vs* 27%) and antianaemics (10 *vs* 4%), both more frequent in the cisplatin arm (Table 4

**Table 4:** Does cisplatin-based chemotherapy affect SC?

	**Cisplatin-based (*N*=181)**	**Non-cisplatin (*N*=184)**	***P*^a^**
*Drugs against treatment side effects*	*120 (66%)*	*113 (61%)*	*0.33*
A01 Stomatologicals	0	0	
A02 Antacids	39 (22%)	39 (21%)	
A03 Antispasmodics	28 (15%)	31 (17%)	
A04 Antiemetics	75 (41%)	49 (27%)	
A06 Laxatives	14 (8%)	18 (10%)	
A07 Antidiarrhoeals	9 (5%)	6 (3%)	
B02 Antihaemorrhagics	2 (1%)	10 (5%)	
B03 Antianaemics	19 (10%)	8 (4%)	
J01 Antibacterials for systemic use	15 (8%)	18 (10%)	
J02 Antimycotics for systemic use	4 (2%)	4 (2%)	
L03AA Colony-stimulating factors	23 (13%)	19 (10%)	

*Drugs against tumour symptoms*	*104 (57%)*	*98 (53%)*	*0.42*
A14 Anabolic agents for systemic use	2 (1%)	2 (1%)	
A15 Appetite stimulants	0	0	
B01 Antithrombotic agents	7 (4%)	3 (2%)	
H01AA ACTH	2 (1%)	3 (2%)	
H02 Corticosteroids for systemic use	70 (39%)	61 (33%)	
L02AB Progestogens	3 (2%)	0	
M01A Nonsteroidal anti-inflammatory drugs	38 (21%)	26 (14%)	
M05B Drugs affecting mineralisation	8 (4%)	5 (3%)	
N02A Opioids	19 (10%)	10 (5%)	
N02B Other analgesics and antipyretics	18 (10%)	21 (11%)	
N03 Antiepileptics	0	2 (1%)	
R03 Antiasthmatics	9 (5%)	15 (8%)	
R05C Expettorants	4 (2%)	3 (2%)	
R05D Cough suppressants	9 (5%)	11 (6%)	

*Drugs against concomitant diseases*	*20 (11%)*	*12 (7%)*	*0.13*
A10 Antidiabetics	4 (2%)	1 (1%)	
C Cardiovascular system	16 (9%)	5 (3%)	
G04 Drugs for prostatic hypertrophy	0	1 (1%)	
H03 Drugs for thyroid	0	1 (1%)	
N05 Psycholeptics	2 (1%)	2 (1%)	
N06 Psychoanaleptics	1 (2%)	2 (1%)	
N07C Antivertigo preparations	0	0	

Adult Italian patients randomized in the GEMVIN3 study. The table shows the number (percentage) of patients assuming at least one drug of each category during the first 63 days of treatment

a *χ*^2^ test.

).

### Does PS affect SC?

In the whole series of 1185 patients, 77% of PS 0 and 78% of PS 1 patients received three or more cycles of chemotherapy, as compared to 60% of those with PS 2. This striking difference, possibly due to worse prognosis or reduced tolerance to chemotherapy, leads to underestimation of the amount of supportive drugs consumed by PS 2 patients because of the shorter exposure time. Thus, analysis of the effect of PS on SC was limited to the 883 patients (759 with PS 0–1 and 124 with PS 2) who had fulfilled all the three first cycles: the analysis is still conservative because of the exclusion of the worst performing patients, more frequent among those with PS 2.

Overall, 666 out of 883 patients (75%) assumed at least one supportive drug. The mean number of supportive drugs assumed by PS 0–1 patients was 2.3 as compared with 2.5 in the PS 2 patients (*P*=0.41). Drugs against side effects of treatment were assumed by 53 *vs* 58% (*P*=0.24), drugs against symptoms by 56 *vs* 62% (*P*=0.28), drugs against concomitant diseases by 15 and 23% (*P*=0.10), in patients with better and worse PS, respectively. Patients with worse PS received antiacids and corticosteroids much more frequently than PS 0–1 patients (Table 5

**Table 5:** Does performance status (PS) affect SC?

	**PS 0/1 (*N*=759)**	**PS 2 (*N*=124)**	***P*^a^**
*Drugs against treatment side effects*	*406 (53%)*	*72 (58%)*	*0.24*
A01 Stomatologicals	8 (1%)	1 (1%)	
A02 Antacids	141 (19%)	33 (27%)	
A03 Antispasmodics	99 (13%)	11 (9%)	
A04 Antiemetics	141 (19%)	17 (14%)	
A06 Laxatives	73 (10%)	17 (14%)	
A07 Antidiarrhoeals	20 (3%)	2 (2%)	
B02 Antihaemorrhagics	27 (4%)	5 (4%)	
B03 Antianaemics	41 (5%)	5 (4%)	
J01 Antibacterials for systemic use	68 (9%)	8 (6%)	
J02 Antimycotics for systemic use	13 (2%)	2 (2%)	
L03AA Colony-stimulating factors	71 (9%)	7 (6%)	

*Drugs against tumour symptoms*	*424 (56%)*	*77 (62%)*	*0.28*
A14 Anabolic agents for systemic use	8 (1%)	0	
A15 Appetite stimulants	1 (<1%)	0	
B01 Antithrombotic agents	28 (4%)	0	
H01AA ACTH	26 (3%)	7 (6%)	
H02 Corticosteroids for systemic use	230 (30%)	52 (42%)	
L02AB Progestogens	11 (1%)	1 (1%)	
M01A Nonsteroidal anti-inflammatory drugs	122 (16%)	24 (19%)	
M05B Drugs affecting mineralisation	18 (2%)	4 (3%)	
N02A Opioids	64 (8%)	13 (10%)	
N02B Other analgesics and antipyretics	97 (13%)	20 (16%)	
N03 Antiepileptics	4 (1%)	0	
R03 Antiasthmatics	61 (8%)	9 (7%)	
R05C Expettorants	27 (4%)	5 (4%)	
R05D Cough suppressants	58 (8%)	14 (11%)	

*Drugs against concomitant diseases*	*116 (15%)*	*28 (23%)*	*0.10*
A10 Antidiabetics	12 (2%)	0	
C Cardiovascular system	80 (11%)	20 (16%)	
G04 Drugs for prostatic hypertrophy	2 (<1%)	0	
H03 Drugs for thyroid	2 (<1%)	1 (1%)	
N05 Psycholeptics	22 (3%)	5 (4%)	
N06 Psychoanaleptics	9 (1%)	2 (2%)	
N07C Antivertigo preparations	2 (<1%)	0	

Patients with PS 0/1 *vs* patients with PS 2 in all three studies. The table shows the number (percentage) of patients assuming at least one drug of each category during the first 63 days of treatment. Only patients receiving three or more cycles of chemotherapy are considered.

a Mantel Haenszel test stratified by treatment arm.

).

### Does age affect SC?

In order to avoid bias related to different chemotherapy, impact of age on SC was studied by comparing 184 adult (<70 years) *vs* 219 elderly (⩾70 years) patients treated with the same chemotherapy (gemcitabine plus vinorelbine in the MILES and GEMVIN3 studies). Overall, 306 out of 403 patients (76%) received at least one supportive drug. The mean number of supportive drugs assumed by adult patients was 2.2, and that in the elderly patients 2.3 (*P*=0.75). As shown in Table 6

**Table 6:** Does age affect SC?

	**Adult patients (*N*=184)**	**Elderly patients (*N*=219)**	***P*^a^**
*Drugs against treatment side effects*	*113 (61%)*	*120 (55%)*	*0.22*
A01 Stomatologicals	0	4 (2%)	
A02 Antacids	39 (21%)	41 (19%)	
A03 Antispasmodics	31 (17%)	27 (12%)	
A04 Antiemetics	49 (27%)	26 (12%)	
A06 Laxatives	18 (10%)	29 (13%)	
A07 Antidiarrhoeals	6 (3%)	7 (3%)	
B02 Antihaemorrhagics	10 (5%)	7 (3%)	
B03 Antianaemics	8 (4%)	12 (5%)	
J01 Antibacterials for systemic use	18 (10%)	21 (10%)	
J02 Antimycotics for systemic use	4 (2%)	3 (1%)	
L03AA Colony-stimulating factors	19 (10%)	22 (10%)	

*Drugs against tumour symptoms*	*98 (53%)*	*113 (52%)*	*0.76*
A14 Anabolic agents for systemic use	2 (1%)	0	
A15 Appetite stimulants	0	0	
B01 Antithrombotic agents	3 (2%)	8 (4%)	
H01AA ACTH	3 (2%)	14 (6%)	
H02 Corticosteroids for systemic use	61 (33%)	60 (27%)	
L02AB Progestogens	0	3 (1%)	
M01 A Nonsteroidal anti-inflammatory drugs	26 (14%)	28 (13%)	
M05B Drugs affecting mineralisation	5 (3%)	5 (2%)	
N02A Opioids	10 (5%)	20 (9%)	
N02B Other analgesics and antipyretics	21(11%)	26 (12%)	
N03 Antiepileptics	2 (1%)	1 (<1%)	
R03 Antiasthmatics	15 (8%)	15 (7%)	
R05C Expettorants	3 (2%)	3 (1%)	
R05D Cough suppressants	11 (6%)	16 (7%)	

*Drugs against concomitant diseases*	*12 (7%)*	*44 (20%)*	*0.0001*
A10 Antidiabetics	1 (1%)	1 (<1%)	
C Cardiovascular system	5 (3%)	35 (16%)	
G04 Drugs for benign prostatic hypertrophy	1 (1%)	1 (<1%)	
H03 Drugs for thyroid	1 (1%)	1 (<1%)	
N05 Psycholeptics	2 (1%)	6 (3%)	
N06 Psychoanaleptics	2 (1%)	2 (1%)	
N07C Antivertigo preparations	0	1 (<1%)	

Patients receiving gemcitabine plus vinorelbine in the GEMVIN3 study (adult patients) *vs* those receiving the same chemotherapy in the MILES study (elderly patients). The table shows the number (percentage) of patients assuming at least one drug of each category during the first 63 days of treatment.

a Mantel–Haenszel test stratified by PS category.

, drugs against treatment side effects were assumed by 61 *vs* 55% (*P*=0.22), drugs against symptoms by 53 *vs* 52% (*P*=0.76), drugs against concomitant diseases by 7 *vs* 20% (*P*<0.0001), in adults and elderly patients, respectively. Among drugs against toxicity, use of antiemetics was more frequent among adults than elderly people (27 *vs* 12%). Among drugs for concomitant diseases, cardiovascular drugs were more frequently used in elderly than adults (16 *vs* 3%).

## DISCUSSION

A significant proportion of the patients included in the present analysis assumed three or more different drugs in addition to chemotherapy. Polypharmacotherapy, defined as the simultaneous assumption of many drugs, can produce noxious effects (Alderman, 2000). Among the several problems related to polypharmacotherapy, one of the most frequently addressed is the higher number of adverse drug reactions and drug–drug interactions, which can become crucial with drugs with a narrow therapeutic index, that is, small difference between therapeutic and toxic doses. Another problem is treatment compliance; the more drugs a patient takes, the harder it is to keep their administration correct. For example, in a study of patients with either diabetes or congestive heart failure, among patients taking one drug, 15% made errors, while those taking two or three drugs had a 25% error rate and over 35% of those taking four or more drugs made errors (Hulka *et al*, 1975). In a large study analysing the causes of medical emergencies in elderly people (Malhotra *et al*, 2001), there was a significant contribution of adverse drug reactions, accounting for 6.7% of the events, and noncompliance with medications, accounting for another 7.6%. Polypharmacotherapy was associated with an increased risk of admission both for adverse reactions and noncompliance. Last but not least, pharmacoeconomic considerations should not be forgotten, especially when prescribing drugs characterised by high costs.

We divided supportive drugs into three categories: drugs against treatment side effects, against tumour symptoms and against concomitant diseases. More than half of the patients assumed drugs of the first two groups, while one-fifth assumed drugs against concomitant diseases. Our classification could be debatable for some drugs that can be prescribed with different purposes, for example, antibiotics in the SC arm of the ELVIS trial (see Table 3) that were probably prescribed to counteract infectious episodes typical of chronic obstructive pulmonary disease frequently associated with lung cancer.

Studying factors affecting the use of supportive drugs we found four main results: (i) a relatively low-toxic chemotherapeutic agent (vinorelbine) does not produce substantial variations in the SC pattern; (ii) a more toxic cisplatin-based treatment requires an overall higher number of supportive drugs, and exposes a higher rate of patients to antiemetics and antianaemics; (iii) a deteriorated performance status is associated with an increased exposure to corticosteroids and a general tendency to an increased use of supportive drugs; (iv) elderly patients require drugs against concomitant diseases significantly more than adults and are less exposed to antiemetics.

As for the first two points, it is a common opinion that side effects of chemotherapy impair quality of life and require assumption of drugs against toxicity. Impact of chemotherapy on quality of life was ruled out by the primary analysis of the ELVIS study showing an overall improvement of quality of life (The ELVIS group, 1999), and it appears now that there are no statistically significant differences in the SC pattern, with the obvious exception on CSFs, not used in the SC arm. The similar rate of patients exposed to antiacid in the two arms is clearly related to the prevention of gastric side effects of corticosteroids, assumed by about half of the patients. Of course, this result is driven by the use of single agent vinorelbine, a drug known for its good tolerability and the relatively low incidence of side effects. In fact, a difference in the mean number of drugs assumed by the patients was found between the arm receiving cisplatin-based chemotherapy and the arm receiving chemotherapy without cisplatin, in the GEMVIN3 trial. The higher incidence of side effects following cisplatin administration (e.g. nausea/vomiting among nonhaematologic toxicities, anaemia among haematologic toxicities) produced higher assumption of several drug categories (e.g. antiemetics and antianaemic preparations) when compared to the combination of two less toxic drugs, gemcitabine and vinorelbine.

Performance status has an important prognostic role in patients with advanced NSCLC (Ando *et al*, 2001). A worse PS can be related to pre-existing comorbid conditions, or to pain and other cancer symptoms. Although not reaching a statistically significant difference in the mean number of drugs assumed, a higher intake by patients with worse PS was observed for several drugs against concomitant diseases and cancer symptoms, particularly for corticosteroids. However, our conservative strategy of comparing only patients who actually received three cycles does probably decrease the differences, excluding patients with worse health status among those with PS 2.

As for the impact of age on SC, the higher assumption of drugs for cardiovascular system among elderly patients reflects the higher frequency of comorbidities among these patients (Repetto and Balducci, 2002). The finding that antiemetic agents are more frequently used in younger than older patients, during the same chemotherapy, was unexpected. It is not fully justifiable with incidence of vomiting in the two studies: overall 38% of elderly and 46% of adult patients suffered any grade vomiting, with 8 *vs* 11% suffering grade 2, respectively, and only 1% grade 3 in both groups. With the exception of the higher incidence of acute dystonic reactions in younger patients, age should not significantly predict the incidence of chemotherapy-induced nausea and vomiting or the response to antiemetic treatment. Some studies have shown better control in older patients, whereas others have reported little difference among age groups (Berger and Clark-Snow, 1997). Part of the large difference observed may probably be explained with reluctance in prescribing antiemetics to elderly patients, for whom these drugs could be less manageable and with higher incidence of toxicity.

As this is a secondary analysis of three prospective trials pooled together, some consideration need to be given on the quality of the evidences found. The first two questions (the impact of chemotherapy *vs* SC alone and the impact of cisplatin-based chemotherapy) were each addressed within a specific randomised study; in both of these studies, data on SC were available for most of the patients. Of course, although an *a priori* hypotheses had not been stated and no power calculation had been carried out as for the questions raised in this paper, statistical comparisons presented here can be considered correct, thanks to the randomised design. The two questions regarding the impact of patients' PS and age have been addressed across different randomised studies; thus, they represent indirect explorative subgroup comparisons and their results should be treated with caution. Notwithstanding these limitations, evidences presented here are among the strongest available in the literature. Indeed, descriptions of SC patterns in association with chemotherapy practically do not exist, to the best of our knowledge; in addition, while much interest has been paid to specific drug classes (e.g. antiemetics, CSFs and antibiotics), less attention has been paid to polypharmacotherapy, and to the degree of cytotoxic chemotherapy, and patients' characteristics do affect the overall burden of SC. This is disturbing, considering that oncologists continuously face empiric integration of antineoplastic and supportive drugs. Further studies aimed at a ‘wide-angle’ treatment approach are awaited, which could probably improve our ability of correctly managing cancer patients.

As a final consideration, we believe that three major messages come from our findings: (i) more attention should be paid in clinical practice and research to drug interactions, frequently not well studied and potentially dangerous; (ii) choosing different cytotoxic drugs translates into different levels of cost and drug interaction risk in SC patterns; these consequences should be considered in treatment choice both at singular and population levels; (iii) there are subgroups of patients for whom the issue of SC looks of paramount importance not only because of the limited efficacy of antineoplastic drugs but also for the higher risk of drug interactions. Nevertheless, SC is usually neglected as a matter of research, even in these high-risk patient subgroups.

## APPENDIX

### List of coauthors and participating institutions

National Cancer Institute: Clinical Trials Unit (Francesco Perrone, Massimo Di Maio, Ermelinda De Maio), Medical Oncology B (Cesare Gridelli^1^, Antonio Rossi^1^, Emiddio Barletta, Maria Luisa Barzelloni^2^, Paolo Maione^1^, Rosario Vincenzo Iaffaioli), Naples; Medical Statistics, Second University, Naples (Ciro Gallo, Giuseppe Signoriello); Medical Oncology, S. Carlo Hospital, Potenza (Luigi Manzione, Domenico Bilancia, Angelo Dinota, Gerardo Rosati, Domenico Germano); Monaldi Hospital: Pneumology V (Francovito Piantedosi, Alfredo Lamberti, Vittorio Pontillo, Luigi Brancaccio, Carlo Crispino), Oncology (Alfonso Illiano, Maria Esposito, Ciro Battiloro, Giovanni Tufano), Naples; University Federico II, III Internal Medicine, Naples (Silvio Cigolari^3^, Angela Cioffi, Vincenzo Guardasole, Valentina Angelini, Giovanna Guidetti); Mariano Santo Hospital: Pneumology (Santi Barbera, Francesco Renda, Francesco Romano, Antonio Volpintesta), Medical Oncology, Cosenza; Oncologic Day-Hospital, Civil Hospital, Rovereto (Sergio Federico Robbiati, Mirella Sannicolò); Oncology, Sacco Hospital, Milan (Elena Piazza, Virginio Filipazzi, Gabriella Esani, Anna Gambaro, Sabrina Ferrario); Medical Oncology, Rummo Hospital, Benevento (Giovanni Pietro Ianniello, Vincenza Tinessa, Maria Grazia Caprio); Medical Oncology, S. Paolo Hospital, Milan (Luciano Frontini^4^, Sabrina Zonato, Mary Cabiddu^4^, Alberto Raina^4^); Medical Oncology, S. Maria Goretti Hospital, Latina (Enzo Veltri, Modesto D’Aprile, Giorgio Pistillucci); Medical Oncology, San Lazzaro Hospital, Alba (Federico Castiglione, Gianfranco Porcile, Oliviero Ostellino); Medical Oncology, ULSS 13, Noale (Francesco Rosetti, Orazio Vinante, Giuseppe Azzarello); Oncology, La Maddalena Hospital, Palermo (Vittorio Gebbia, Nicola Borsellino, Antonio Testa); Medical Oncology, Az. Ospedaliera “Bianchi-Melacrino-Morelli”, Reggio Calabria (Giampietro Gasparini^5^, Alessandro Morabito^5^, Domenico Gattuso^5^); Oncology, Cardarelli Hospital, Campobasso (Sante Romito, Francesco Carrozza); Medical Oncology, Civil Hospital, Legnano (Sergio Fava, Anna Calcagno, Emanuela Grimi); Medical Oncology, Molinette Hospital, Turin (Oscar Bertetto, Libero Ciuffreda, Giuseppe Parello); Medical Oncology, San Gennaro Hospital, Naples (Luigi Maiorino, Antonio Santoro, Massimiliano Santoro); Medical Oncology, S. Luigi and SS. Currò Gonzaga Hospital, Catania (Giuseppe Failla, Rosa Anna Aiello); Medical Oncology, CRO, Aviano (Alessandra Bearz, Roberto Sorio, Simona Scalone); Medical Oncology, S. Giuseppe Hospital, Milan (Maurizia Clerici, Roberto Bollina, Paolo Belloni); Medical Oncology, S. Maria della Misericordia Hospital, Udine (Cosimo Sacco, Angela Sibau); Medical Oncology, University, Messina (Vincenzo Adamo, Giuseppe Altavilla, Antonino Scimone); Pneumology, University, Palermo (Mario Spatafora, Vincenzo Bellia, Maria Raffaella Hopps); Medical Oncology, Civil Hospital, Padova (Silvio Monfardini, Adolfo Favaretto, Micaela Stefani); Medical Oncology, USSL 33, Rho (Giuliana Mara Corradini, Gianfranco Pavia); Pneumology, S. Luigi Gonzaga Hospital, Orbassano (Giorgio Scagliotti, Silvia Novello, Giovanni Selvaggi); Medical Oncology, University, Perugia (Maurizio Tonato, Samir Darwish); Ospedali Riuniti: Pneumology (Giovanni Michetti, Maria Ori Belometti), Medical Oncology (Roberto Labianca, Antonello Quadri), Bergamo; Pneumooncology, Forlanini Hospital, Roma (Filippo De Marinis, Maria Rita Migliorino, Olga Martelli); Experimental Medical Oncology, Oncologic Institute, Bari (Giuseppe Colucci, Domenico Galetta, Francesco Giotta); Oncology, Serbelloni Hospital, Gorgonzola (Luciano Isa, Paola Candido); Oncology, Civil Hospital, Polla (Nestore Rossi, Antonio Calandriello); Medical Oncology, S. Vincenzo Hospital, Taormina (Francesco Ferraù, Emilia Malaponte); Medical Oncology, Civil Hospital, Treviglio (Sandro Barni, Marina Cazzaniga); Chemotherapy, University, Palermo (Nicola Gebbia, Maria Rosaria Valerio); Medical Oncology, Civil Hospital, Avellino (Mario Belli, Giuseppe Colantuoni); Thoracic Surgery, University, Foggia (Matteo Antonio Capuano, Michele Angiolillo, Francesco Sollitto); Oncologic Radiotherapy, S. Gerardo Hospital, Monza (Antonio Ardizzoia); Medical Oncology, S. Carlo Borromeo Hospital, Milan (Gino Luporini, Maria Cristina Locatelli); Oncology−Hematology, C. Poma Hospital, Mantova (Franca Pari, Enrico Aitini); Oncology, Fatebenefratelli Hospital, Benevento (Tonino Pedicini, Antonio Febbraro, Cesira Zollo); Medical Oncology, University, Milano (Paolo Foa^6^); Oncology, S. Maria Hospital, Terni (Francesco Di Costanzo^7^, Roberta Bartolucci, Silvia Gasperoni^7^); Medical Oncology, ULSS 15, Camposampiero (Fernando Gaion, Giovanni Palazzolo); Medical Oncology, S. Chiara Hospital, Trento (Enzo Galligioni, Orazio Caffo); Medical Oncology, University La Sapienza, Rome (Enrico Cortesi, Giuliana D’Auria); Thoracic Surgery, Ascalesi Hospital (Carlo Curcio^8^, Matteo Vasta), Naples; Medical Oncology, S. Giovanni Hospital, Turin (Cesare Bumma, Alfredo Celano, Sergio Bretti^9^); Oncology, Miulli Hospital, Acquaviva delle Fonti (Giuseppe Nettis, Annamaria Anselmo); Medical Oncology, S. Croce Hospital, Fano (Rodolfo Mattioli); Regina Elena Institute: Medical Oncology (Cecilia Nisticò, Annamaria Aschelter), Medical Oncology II, Rome; Medical Oncology, University, Sassari; Pneumology, S. Martino Hospital, Genova; Medical Oncology I, IST, Genova; Oncology, Cottolengo Hospital, Turin; Medical Oncology, S. Bortolo Hospital, Vicenza; Medical Oncology, S. Francesco di Paola Hospital, Paola; Medical Oncology, Centro Catanese di Oncologia, Catania; Oncology, CROB, Rionero in Vulture; Medical Oncology, S. Andrea Hospital, Vercelli; Oncohematology (Medicine I), Maggiore Hospital, Lodi; Medical Oncology, Biomedical Campus, Rome; Oncology, Agnelli Hospital, Pinerolo; Pneumology, S. Corona Hospital, Garbagnate; Medical Oncology, USL 5-Ovest Vicentino; Medical Oncology, G. Di Maria Hospital, Avola; Oncology, S. Paolo Hospital, Bari; Oncology, Civil Hospital, Gorizia; Medical Oncology, Civil Hospital, Nola; Medical Oncology, ASL Lodi, Casalpusterlengo; Medicine, Civil Hospital, Lagonegro; Medical Oncology, Hospital, Lecco; Tisiology and Pneumology, Second University, Monaldi Hospital, Naples; Medical Oncology, University, Businco Hospital, Cagliari; Oncology, Civil Hospital, Sciacca; Medical Oncology, Fondazione Salvatore Maugeri, Pavia; Medical Oncology, Regional Hospital, Bolzano; Businco Oncologic Hospital, Cagliari; Medical Oncology, University, Cagliari; Geriatry, INRCA, Rome; Oncology, Civil Hospital, Ariano Irpino; Oncology, SS. Trinità Hospital, Sora; Pneumology, Galateo Hospital, S. Cesario di Lecce; Medical Oncology, Maggiore Hospital, Trieste; Pneumology, Circolo Varese Hospital, Varese; Medicine, Civil Hospital, Vigevano; Medical Oncology, Casa di Cura Igea, Milan; Tisiology, Policlinico S. Matteo, Pavia; Oncohematology, Pugliese Ciaccio Hospital, Catanzaro; da Procida Hospital: Pneumology, Salerno; Oncology, S. Giovanni di Dio e Ruggi d’Aragona Hospital, Salerno; Geriatric Oncology, Civil Hospital, S. Felice a Cancello; Oncology, C. Cantù Hospital, Abbiategrasso; Thoracic Surgery, Policlinico, Bari; Medical Oncology, Civil Hospital, Legnago; Pneumology, Crema Hospital, Crema; Medical Oncology, USL 1, Sassari; Medical Oncology, Civil Hospital S. Maria delle Grazie, Pozzuoli; Pneumology, Policlinico S. Matteo, Pavia.

**Present addresses:** ^1^S. Giuseppe Moscati Hospital, Avellino; ^2^da Procida Hospital, Salerno; ^3^S. Giovanni di Dio e Ruggi d’Aragona Hospital, Salerno; ^4^Pio X, Milan; ^5^S. Filippo Neri Hospital, Rome; ^6^S. Paolo Hospital, Milan; ^7^Careggi Hospital, Florence; ^8^Monaldi Hospital, Naples; ^9^Civil Hospital, Ivrea.
